# A Review of Supernumerary Teeth in the Premolar Region

**DOI:** 10.1155/2018/6289047

**Published:** 2018-12-03

**Authors:** Khaled Khalaf, Saaid Al Shehadat, Colin A. Murray

**Affiliations:** Department of Preventive and Restorative Dentistry, University of Sharjah, PO Box 27272, UAE

## Abstract

Supernumerary teeth in the premolar region, unlike other supernumeraries, occur more often in the mandible where they are generally of the supplemental type. Occasionally, they are conical or smaller than normal, particularly in the upper premolar regions. They might occur singly or in multiples, be erupted or impacted, but the majority have been found to be unerupted and asymptomatic. The prevalence of supernumerary teeth in the premolar region has been demonstrated to be between 0.01 and 1 percent depending on the population studied. Interestingly, populations from the East or Africa are known to be the most affected. Various theories have been suggested to explain the etiology of supernumerary teeth in general including both genetic and environmental factors. Furthermore, it has been suggested that supernumerary premolar teeth belong to a third (postpermanent) series, developing from extensions of the dental lamina. Several consequences can result from the presence of supernumerary premolars, especially in the mandible, such as cyst formation, transposition, and other clinical scenarios.

## 1. Introduction

There have been multiple review studies reporting on several aspects of supernumerary teeth [1–4] with the majority of these studies being case reports. However, the scientific literature is notably lacking a comprehensive review of supernumerary teeth, particularly in the premolar region. Supernumerary teeth in the premolar region have unique features in comparison with all other supernumeraries in terms of epidemiology and characteristics, etiology, clinical consequences, diagnosis, and management. General dental practitioners (GDP) and dental specialists faced with this not uncommon dental anomaly should have the appropriate knowledge and skills to be able to diagnose and manage patients with this condition safely and effectively. Therefore, the purpose of this review is to provide the general dental practitioner with an update on all aspects of supernumerary teeth in the premolar region to raise their awareness of this dental anomaly so that the most appropriate approach in the diagnosis and management of patients with supernumerary premolars is followed.

## 2. Epidemiology and Characteristics

A literature search was undertaken with PubMed and Ovid Medline databases using the keywords premolar and supernumerary. Relevant publications addressing the prevalence and management of supernumerary premolars were then selected and included in the review. It is relatively common for the occurrence of supernumerary teeth in the premolar region. About 8-9% of all supernumerary teeth occur in the premolar region [1, 4, 5].  Table 1 summarizes nonsyndromic comprehensive studies of supernumerary teeth in the premolar region.

**Figure 1:**
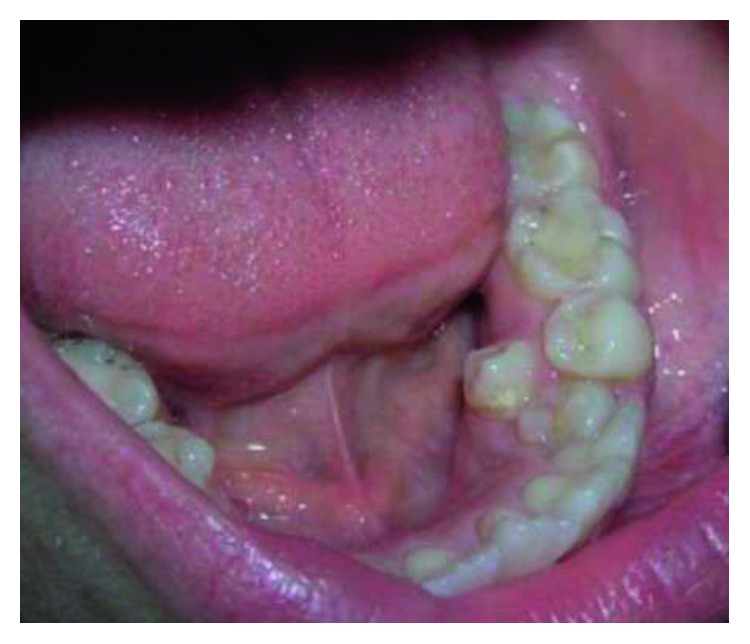
Erupted lower left supernumerary premolar.

**Figure 2:**
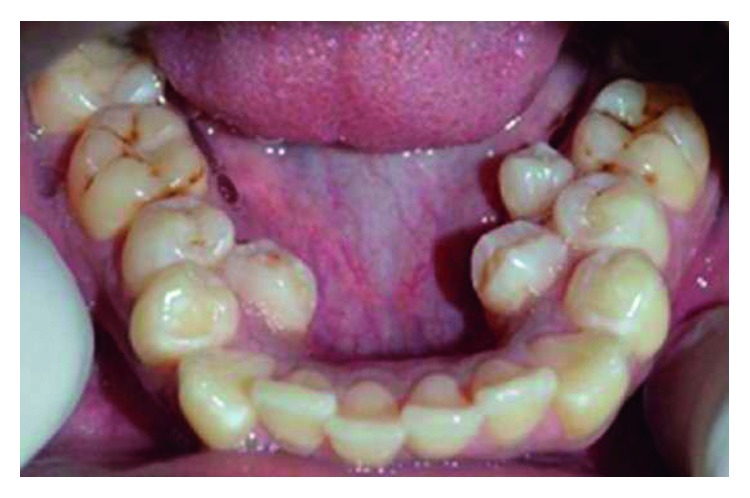
Erupted 3 lower supernumerary premolars.

**Figure 3:**
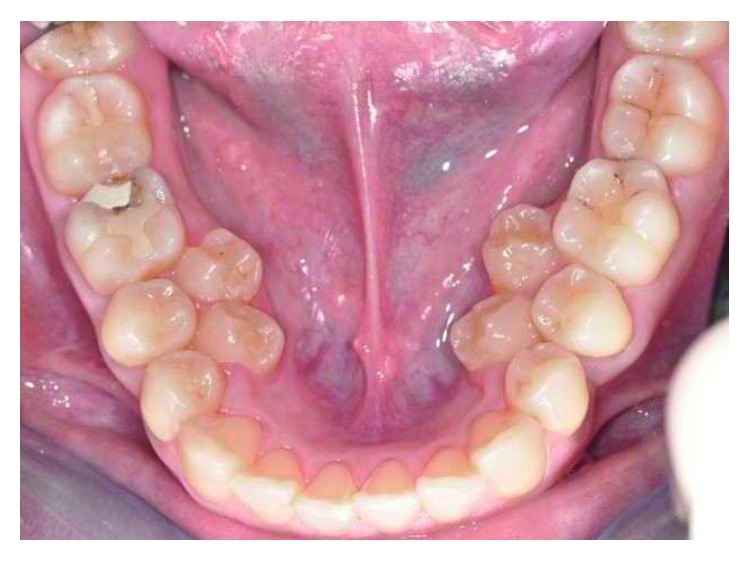
Erupted 4 lower supernumerary premolars.

**Figure 4:**
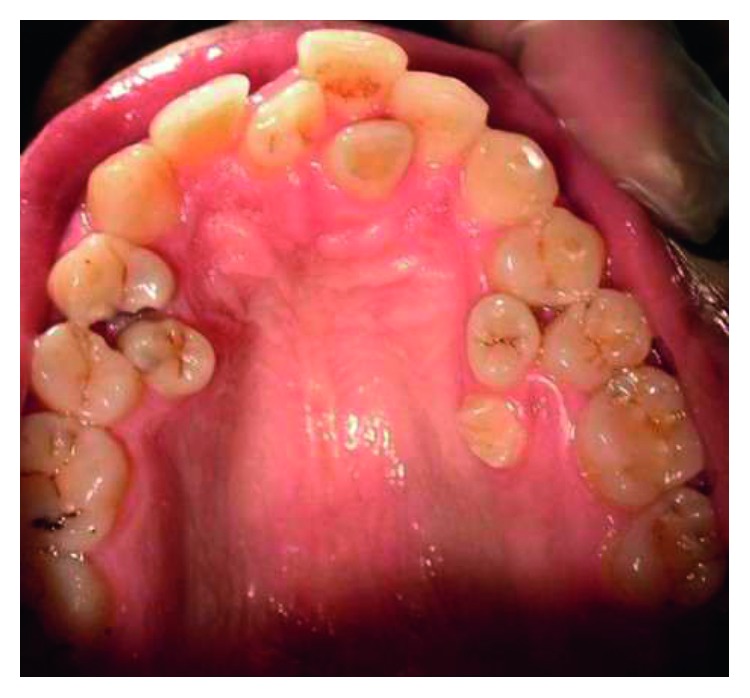
Erupted 3 upper supernumerary premolars associated with additional supernumerary tooth in the upper anterior region.

Supernumerary teeth in the premolar region, unlike other supernumeraries, occur more frequently in the mandible [1, 2], where the supernumerary teeth are generally of the supplemental type (Figures 1–3) [1, 3, 4]. Occasionally, they are conical or smaller than normal particularly in the upper premolar regions (Figure 4) [10–12]. Oehlers [13] stated that supernumerary premolars can be distinguished from those of the normal series as being either diminutive, conical, or, if they are well formed, smaller than normal premolars.

**Figure 5:**
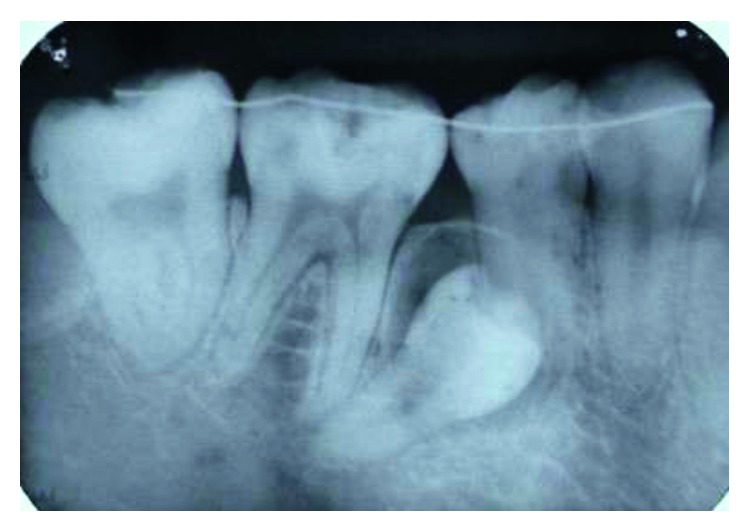
Impacted lower right supernumerary premolar.

Supernumerary teeth in the premolar region might occur singly (Figures 1 and 5) or in multiples (Figures 2–4) [14, 15] and be erupted or impacted (Figure 5).

Seventy-five percent of supernumerary premolars were determined to be unerupted, and the majority of them appeared asymptomatic [16–18]. Thus, a follow-up radiograph is quite useful for orthodontic patients to detect any unerupted supernumerary premolars that might have an effect throughout the treatment. The mandibular premolar region was found to have the highest frequency of supernumerary teeth in the condition “nonsyndrome multiple supernumerary teeth” [19]. Stafne [1] found that 8.4 percent of all supernumerary teeth were in the premolar region, with 6.6 percent of the total in the mandible. A similar figure (8.0%) has been given by Nazif et al. [4], whereas Grahnen and Lindahl [5] reported that supernumerary premolars represent 9.1 percent of all supernumerary teeth.

From the literature, it is evident that most of the supernumerary teeth reported in the premolar region have been found in patients from the East or from Africa. Still [3] stated that, in Southern Nigeria, approximately 1 percent of the population has one or more supernumerary premolar teeth.

Poyton et al. [20] reported the incidence of supernumerary premolars as 0.01% and 1% in 10,000 persons. They established that 75 percent of supernumerary premolars were impacted and generally unerupted. A higher incidence (0.29% of general populations) has been reported by Grahnen and Lindahl [5] in Swedish dental students. Similarly, Kaya et al. [8] have reported the presence of supernumerary premolars in 10 out of 8400 patients (0.24%). Whereas in an orthodontic population, the frequency of supernumerary premolars was as high as 0.64% (7 in 1,100) and the age range of the patients when detecting the supernumerary premolars was between eleven and sixteen years [7].

Six patients (0.15%) were found to have supernumerary mandibular premolars in a sample population of 4,000 patient records [6]. Four cases were from a suburban private practice sample of 2,000, and the other 2 cases were from a similar-sized group, randomly drawn from an urban dental school clinic. The male-to-female ratio in this study was five to one. Four were of Hispanic descent, one was black, and one was white. This again supports the theory that supernumerary premolars are more common in races other than white. Seven supernumerary premolars were found in those six patients, five appeared on the left side, and six were impacted or erupted ectopically. The only other supernumerary teeth seen in this sample were two maxillary fourth molars and one mesiodens. The figures found in this study were not in agreement with what has been reported by many authors, neither in the frequency [1, 4, 21, 22] nor in the order of the affected teeth [1, 21, 23–26]. The small sample size in this study could possibly explain the difference found and emphasize the use of sufficient numbers to have comparative figures.

## 3. Etiology

**Figure 6:**
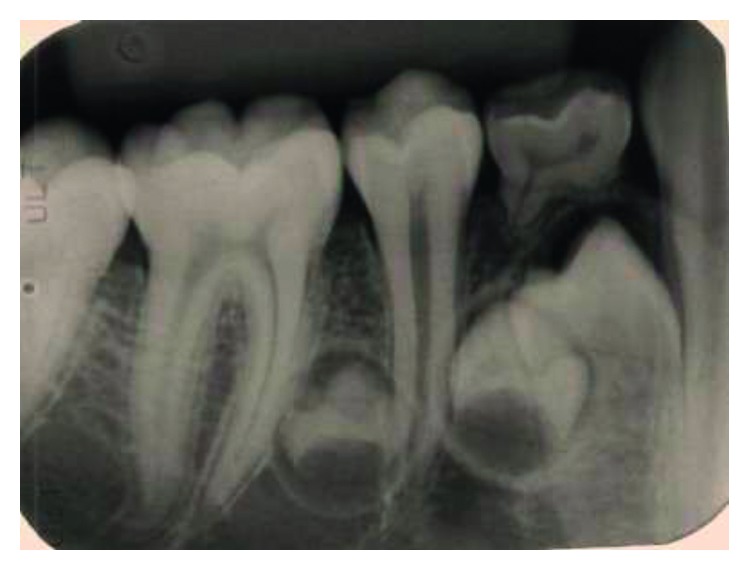
Two late-forming supernumerary premolars.

Various theories have been suggested to explain the etiology of supernumerary teeth including both genetic and environmental factors [27, 28]. Furthermore, it has been suggested that supernumerary premolar teeth belong to a third (postpermanent) series, developing from extensions of the dental lamina [11, 13, 20, 29]. This has been supported by the common finding of the incomplete root formation of these teeth in comparison with that of the normal premolars, which appear to have complete root formation (Figure 6) [1, 7, 11, 13, 14, 18, 29–34].

Moreover, Price and Hoggins [11] established that the development of supernumerary premolars, estimated from their own 5 cases and those of previous authors, occurred 7 to 10 years later than premolar teeth of the normal series. Also, they explained the different stages of development of the supernumerary premolars found by some authors [20] as indication of a new generation of premolar teeth, the postpermanent and post-postpermanent dentitions, with a difference of 5 years between them. Similar figures of estimated time periods of development of the supernumerary premolars have been given by Bowden [29] (7.5 to 11 years after the development of the normal series), Kantor et al. [32], and Rubenstein et al. [7] (approximately 7–11 years after normal development). Various cases have shown that supernumeraries in the mandibular premolar region were first visible at the ages of nine, twelve to twelve and a half, thirteen and a half, and fourteen years [16–18, 33, 35]. However, it is difficult to determine exactly when a supernumerary tooth starts to form due to the difficulty of their detection on routine radiographs as they are commonly found in the lingual position. Timing for the development of supernumerary premolar roots is still controversial. However, Oehlers [13] reported a continued root growth of supernumerary premolars in a twenty-three-year-old man.

Ranta and Ylipaavalniemi [31] reported 2 cases of supernumerary mandibular premolars that developed coincidently after the occurrence of jaw fractures, with one of them observed over an 11-year period. At age 11.1 years, during the treatment of mandibular fractures, there were no signs of supernumerary teeth. At age 15 years, three supernumeraries were present in the mandible (with mineralization stages, crown, one-fourth to three-fourths completed) with chronological ages of approximately four to six years. At age 16.7 years, further development was observed with mineralization approximated to a chronological age of six to seven years. At age 22 years, all supernumeraries reported were still present, with almost complete root formation and a chronological age of 13 to 14 years. Many other studies have also reported late-developing supernumerary teeth in the premolar region [18, 29, 32, 34, 36–38].

Bowden [29] reported 3 cases of tuberculate maxillary supernumerary teeth in addition to supernumerary premolar teeth. From these 3 cases (all taken from the case files of orthodontists) and others [39, 40], the tuberculate supernumerary tooth is usually close to the upper central incisor, in the palatal position, with root formation later in development than either the permanent central incisor or the conical supernumerary tooth. Failure of eruption of the adjacent teeth often occurs. Thus, it seems that these different types of supernumerary teeth (maxillary tuberculate and premolar teeth) develop at times not only relevant to the normal series of the same region but also to each other. This may support the theory that both these types of supernumerary teeth are units of the same, new generation of the dentition, the third or postpermanent dentition.

## 4. Consequences/Sequelae

**Figure 7:**
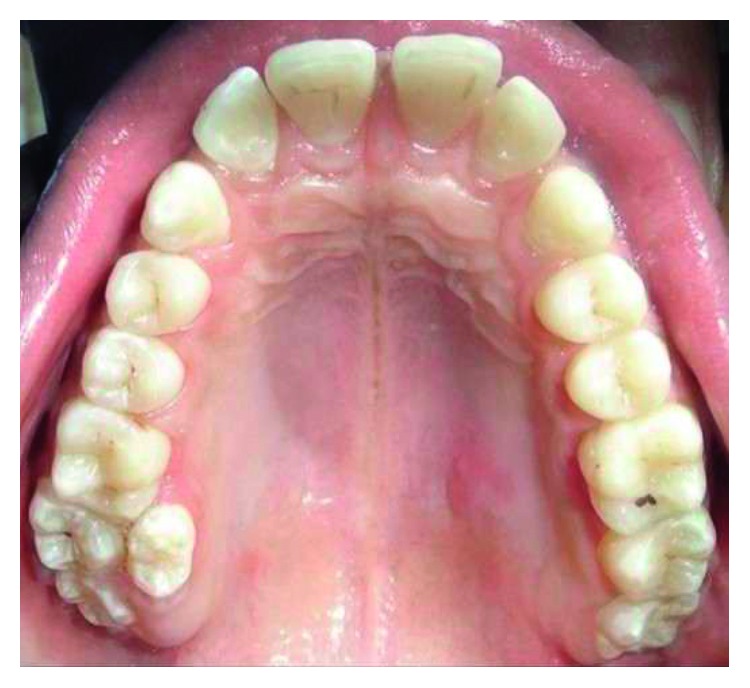
Erupted supernumerary premolar in the upper right molars region.

Mandibular supernumerary premolars have a marked tendency towards the formation of cysts and pathological changes [20, 41, 42]. Transposed supernumerary premolars in occlusion have also been reported [15, 34, 43, 44]. However, in cases where supernumerary teeth of premolar form are found in the molar regions (Figure 7), it is hard to determine whether these supernumerary teeth belong to the premolar normal series in origin and are for some reason transposed to the molar area, or originally arise from the region where they are found, with a form similar to that of premolars. Supernumerary teeth that originally belong to the molar region are mostly found between the second and third molars (paramolars) or behind the third molars (fourth molars). Therefore, in addition to other characteristics of supernumeraries in different regions, this might be helpful as a diagnostic sign for differentiation between supernumerary teeth in the molar region and transposed supernumerary premolars. Different cases of supernumerary premolar teeth in the molar region have been considered as transposed supernumerary premolars [15, 34, 45].

Late-developing supernumerary teeth in the premolar region are often detected at the end of orthodontic treatment at approximately 13 years of age and may interfere with space closure and implant placement [46]. Furthermore, cases of fusion between supernumerary premolars and the adjacent teeth have been reported [47, 48].

## 5. Diagnosis and Management

Early diagnosis of supernumerary teeth in the premolar region is important in their management. This should involve taking a thorough medical and dental history and carrying out a radiological assessment following a clinical examination. Most supernumerary teeth in the premolar region are unerupted, with no clinical signs or symptoms and often observed as incidental findings on radiographs [7, 15, 16, 34]. Furthermore, supernumerary teeth in the premolar region often develop late and frequently after the start of orthodontic treatment [7, 18], or they may recur after their removal. Therefore, the role of radiological assessment for all new patients should be emphasized upon [1, 6, 16, 34, 49, 50]. The most appropriate radiographic view is a panoramic radiograph supplemented with periapicals as required, as unerupted supernumerary premolar teeth normally develop lingually and apically to the normal series [7, 29]. Thus, a first panoramic view is required prior to the start of orthodontic treatment to make a definitive diagnosis of the presence/absence of anomalies of tooth number, and another one may be required later on during treatment to rule out development of any new supernumeraries and prevent any adverse effects this may have on the progress of orthodontic treatment or any iatrogenic effects.

A decision needs to be made whether to extract or leave supernumerary premolar teeth in situ once they are diagnosed [38, 51]. If a supernumerary premolar is erupted in a reasonable alignment and with no consequences on the occlusion, then it can be left in situ. However, if the supernumerary premolar tooth has erupted/is erupting with a lack of space, then early removal should be undertaken to relieve crowding and/or prevent occlusal discrepancies [52, 53]. If a supernumerary premolar tooth is unerupted, as occurs in the majority of patients, and diagnosed early, then early removal prior to commencing orthodontic treatment is often recommended [7, 11]. Conversely, others recommend delaying removal of the supernumerary premolars until further root development of the supernumerary teeth have occurred [6, 12, 15, 34, 54] or until the full permanent dentition has been established [55] to avoid any iatrogenic damage to neighbouring anatomical structures and roots of the normal teeth. In the case of late-developing supernumerary teeth in the premolar region, the management options will be either to extract the supernumerary tooth or to monitor it by means of regular radiographic and clinical follow-up depending on the time of appearance of the supernumerary tooth. If it has appeared prior to or at the early stage of orthodontic treatment and it is likely to interfere with orthodontic treatment, then it will be recommended to extract it. Otherwise, if it appears at the end of orthodontic treatment without disturbing the occlusion, it is recommended to monitor it [38].

Ultimately, it is the clinician's responsibility to fully explain all appropriate and feasible treatment options to the patient and/or guardian including the risks and benefits of each option as part of the consent process. The patient and/or guardian's wishes should be respected as to which treatment option should be chosen in each case. The patient and/or guardian may choose to leave any unerupted supernumerary premolar tooth in situ, especially if it has not caused any iatrogenic effects or is unlikely to interfere with orthodontic treatment. In such a case, it is important to highlight to the patient and/or guardian the potential risks that may occur in the future and the need for periodic radiographic monitoring of the unerupted supernumerary premolar tooth/teeth to detect any iatrogenic damage at an early stage which may occur [56].

## 6. Conclusions

1. Supernumerary teeth in the premolar region, unlike other supernumeraries, occur more often in the mandible, where the supernumerary teeth are generally of the supplemental type.
2. They might occur singly or in multiples, be erupted or impacted, but the majority is found to be unerupted and asymptomatic.
3. The prevalence of supernumerary teeth in the premolar region is between 0.01 and 1 percent depending on the population studied, with populations from the East or Africa the most affected.
4. It has been suggested that supernumerary premolar teeth belong to a third (postpermanent) series, developing from extensions of the dental lamina.
5. Several consequences can result from the presence of supernumerary premolars, especially in the mandible, such as cyst formation, transposition, and malocclusion.

## Figures and Tables

**Table 1:** Summary of nonsyndromic comprehensive studies of supernumerary teeth in the premolar region.

Authors	Year	Country	Sample	No. of cases	No. of teeth	Prevalence %	Maxilla	Mandible
Stafne [1]	1932	USA	48,550	NR^*∗*^	42	0.084	9	33
Grahnen and Lindhal [5]	1961	Sweden	1,052	3	6	0.29	0	6
Zvolanek and Spotts [6]	1985	USA	4,000	6	7	0.15	0	7
Rubenstein et al. [7]	1991	USA	1,100	7	16	0.64	3	13
Kaya et al. [8]	2011	Turkey	8400	10	20	0.24	1	19
Arikan et al. [9]	2013	Turkey	7,551	13	13	0.17	0	13

^*∗*^NR = not reported.
